# Beyond the peak: A deterministic compartment model for exploring the Covid-19 evolution in Italy

**DOI:** 10.1371/journal.pone.0241951

**Published:** 2020-11-06

**Authors:** Silvio Romano, Annalisa Fierro, Antonella Liccardo

**Affiliations:** 1 Physics Department, Università degli Studi di Napoli “Federico II”, Napoli, Italy; 2 CNR-SPIN, Napoli, Italy; Columbia University, UNITED STATES

## Abstract

Novel Covid-19 has had a huge impact on the world’s population since December 2019. The very rapid spreading of the virus worldwide, with its heavy toll of death and overload of the healthcare systems, induced the scientific community to focus on understanding, monitoring and foreseeing the epidemic evolution, weighing up the impact of different containment measures. An immense literature was produced in few months. Many papers were focused on predicting the peak features through a variety of different models. In the present paper, combining the surveillance data-set with data on mobility and testing, we develop a deterministic compartment model aimed at performing a retrospective analysis to understand the main modifications occurred to the characteristic parameters that regulate the epidemic spreading. We find that, besides self-protective behaviors, a reduction of susceptibility should have occurred in order to explain the fast descent of the epidemic after the peak. A sensitivity analysis of the basic reproduction number, in response to variations of the epidemiological parameters that can be influenced by policy-makers, shows the primary importance of a rigid isolation procedure for the diagnosed cases, combined with an intensive effort in performing extended testing campaigns. Future scenarios depend on the ability to protect the population from the injection of new cases from abroad, and to pursue in applying rigid self-protective measures.

## Introduction

On 20th February 2020 the first local transmission case of the novel coronavirus Covid-19 [1], recently causing a dramatic outbreak in Wuhan City, Hubei Province, China, [2–5] was confirmed in Italy. Even if at the end of January Covid-19 was already declared as a Public Health Emergency of International Concern from the WHO, the awareness, that the outbreak started to have its own life within other countries, frightened the whole world.

The intensive research of the Italian Patient Zero opened a Pandora’s box, leading to the identification of more than 1000 cases in 1 week, and thus revealing that the disease was already circulating at least since January, in spite of the effective measures of travel restrictions from China. In order to contain the outbreak, severe mobility restrictions were soon implemented in the Italian area where the epidemic firstly appeared (Lombardia, Veneto, Emilia Romagna), and then extended to the whole country [6].

In such an emergency context, epidemiologists were expected to get ahead of the epidemic, although its features were in rapid evolution, and to provide guidance to the local governments, by predicting future scenarios and weighing up the effect of possible mitigation strategies [7–11]. The major challenge that epidemiologists had to face was double-fold: on one hand they had to work with real-time data affected by unavoidable under-reporting bias and subject to frequent adjustments, on the other hand they had to construct predictive models within an epidemic scenario that was changing day by day because of containment strategies, self-protective measures adopted by individuals as a consequence of increased risk perception, saturation of the health care system, different treatment protocols or modifications in the strength of the virus.

The urgency to give answers to the policy makers and to the public opinion led to a frenetic activity of the scientific community, with the consequent production of a huge literature in a relatively short period. Many papers made predictions about the peak features, in particular when it would have been reached and with how many infections [12, 13], the final size, duration and attack rate of the outbreak [14]. At the 1st of August the scenario in Italy was definitely worse than in early predictions: more than 250 thousand detected infections, and almost 35 thousand deaths [15].

Many of the published models are conceived as simple extensions of the deterministic SIR model [16]. The mean field approach is at the base of a number of papers discussing the Chinese case, as for instance the SEIR metapopulation model of [17], the SEIR model with the inclusion of individual behavior reaction in [18], the *θ*-SEHIRD model of [19], that focuses on the role of infectious undetected cases.

The adequacy of a simple SIR model to obtain quantitative predictions on the Covid-19 evolution is asserted in many recent papers [5, 20–24]. In [12], authors analyze the epidemiological surveillance data from different countries affected by the Covid-19 pandemic, and study day by day maps for cumulative confirmed cases, recovered people and cumulative reported deaths, finding that the analyzed data follow the same power law behavior. The existence of an universality in the epidemic spreading in different countries is interpreted as a confirmation that a mean-field approach may be useful to obtain quantitative information on the epidemic, and specifically the height (how many infected) and time (when) of the epidemic peak.

In [25] a compartment model named SIDARTHE, which includes a finer differentiation of the infected individuals in different compartments (asymptomatic undetected and detected, symptomatic detected and undetected, acutely symptomatic, etc.) is developed for the Italian case. In [26] a metacomunity SEIR model that includes the mobility among different Italian provinces and metropolitan areas is proposed.

In the present paper we wish to make a retrospective analysis in order to understand the modifications occurred to the key features of the epidemic spreading (contact rates, viral loads, risk perceptions, etc.), which allow to reproduce the epidemic curves of the Covid-19 outbreak in Italy from the beginning of the outbreak (20th February 2020) to 1st August 2020. We perform this analysis on a compartment model similar to those already developed in literature, in order to understand the trend of the model parameters after the peak. Each parameter of our model is modulated as an appropriate time dependent function, with a shape that depends on different factors, as for instance the social behavior of individuals, the organization of health-care structures and the mobility restrictions imposed by the Italian government. We find that a significant reduction of the susceptibility parameter is necessary to explain the rapid descent phase of the contagion curve. These modifications are particularly relevant in order to make more reliable previsions about the near future evolution of the pandemic.

We also examine in detail the mathematical dependence of the reproduction number from those epidemiological parameters that can be influenced by the policy makers, showing that a rigid isolation procedure for the diagnosed cases is crucial for the epidemic containment [11]: no matter how extended is the testing campaign, a scarce efficiency in dealing with diagnosed individuals with adequate protection dramatically increase the spreading of the virus. Some future scenarios are also exploited. In particular we find that, in a scenario of fully restored mobility and partially increased susceptibility, is crucial for the population to respect self-protective hygienic measure scrupulously in order to keep the second wave contained within a modest peak, as far as the system may be protected from the injection of new cases from abroad.

## 1 The model

There are some peculiar features of Covid-19 that must be necessarily taken in account in building an epidemiological model for this pandemic.

One of the most challenging aspect to disentangle is the wide variety in the intensity of symptoms among infected individuals. It ranges from severe cases, to mild symptomatic, to pauci-symptomatic and completely asymptomatic individuals. Moreover the mixture among these different categories seems to have undergone an evolution during the epidemic. To follow the evolution of the proportion among different categories, and, eventually understand the reason for such differences in the virus expression, is crucial in order to make reliable predictions on the future spreading of the virus.

Secondly the impressively rapid spread throughout the world of this pandemic has made the diagnostic process and the readiness to treat efficiently infected individuals to be profoundly affected by the healthcare system saturation. In Italy, for instance, since 26th February, only symptomatic individuals were tested against Covid-19, making even more difficult to estimate the fraction of asymptomatic or pauci-symptomatic individuals. On the contrary, in the last weeks of the descendant phase, extensive serological test campaigns were performed allowing to dig up many asymptomatic cases.

From the preliminary results of a serological investigation conducted on the Italian population by ISTAT, Italian Health Ministry and Croce Rossa Italiana, and reported by the media on 3rd August [27], it seems that almost 1.5 millions of individuals, corresponding to 2.5% of the Italian population, have antibodies against Covid-19, signaling that an important fraction of infected individuals remained undetected during the outbreak.

Thus, if it is true that in a typical epidemic outbreak the diagnosed cases (either by serological or swab test) or the Influenza Like Illness (ILI) cases closely follow the trend of infections, and thus can be taken as reliable proxies for the number of infected individuals, in the Covid-19 case such clear relation didn’t hold.

**Fig 1:**
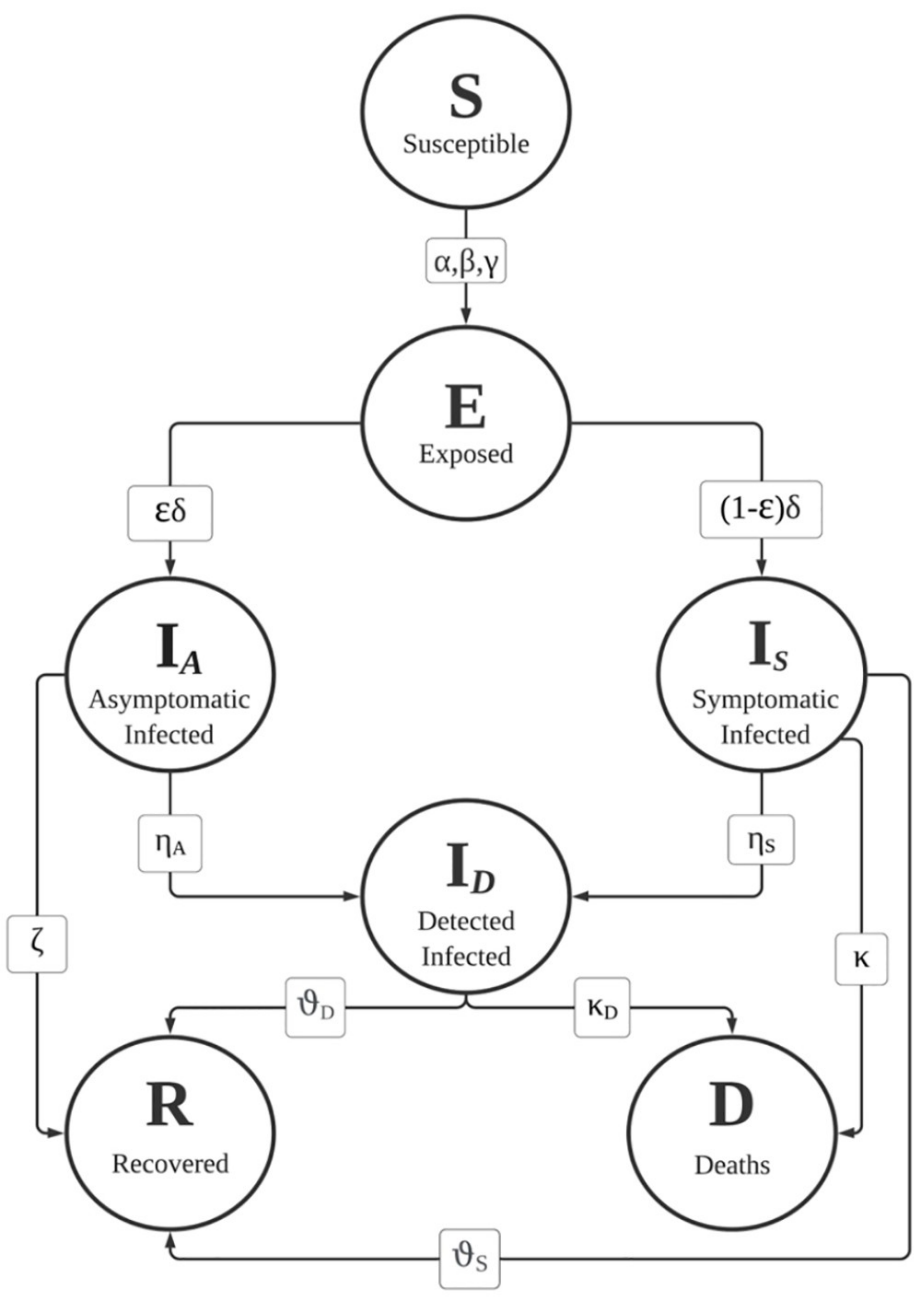
Flow chart summarizing the state variables, fluxes among compartments and related model parameters.

For these reasons, we opt for a model that treats separately symptomatic, asymptomatic and detected individuals. In particular, we build a compartment model, in which individuals are divided in seven mutually exclusive classes according to their epidemiological status: the susceptible compartment *S*(*t*), the exposed compartment *E*(*t*) (i.e. individuals that have been infected but are not yet infective), the asymptomatic infected compartment (that also includes pauci-symptomatic cases) *I*_*A*_(*t*), the symptomatic infective compartment *I*_*S*_(*t*), the diagnosed compartment *I*_*D*_(*t*), and finally the recovered *R*(*t*) and dead *D*(*t*) compartments. We disregard demographic birth and (not related to the virus) death process. The epidemic dynamic is thus governed by the fluxes of individuals among those compartments, as shown in Fig 1, and it is fully described by a system of seven coupled first order differential equations, with 12 parameters for the normalized *SEI*_*A*_ *I*_*S*_ *I*_*D*_ *RD* variables:

$$\begin{array}{c} \left\{ \begin{array}{c} \dot{S}\left(t\right)=-S\left(t\right)\left(\alpha I_{A}\left(t\right)+\beta I_{S}\left(t\right)+\gamma I_{D}\left(t\right)\right)\\ \dot{E}\left(t\right)=S\left(t\right)\left(\alpha I_{A}\left(t\right)+\beta I_{S}\left(t\right)+\gamma I_{D}\left(t\right)\right)-\delta E\\ {\dot{I}}_{A}\left(t\right)=\epsilon \delta E\left(t\right)-\zeta I_{A}\left(t\right)-\eta_{A}I_{A}\left(t\right)\\ {\dot{I}}_{S}\left(t\right)=\left(1-\epsilon \right)\delta E\left(t\right)-\theta_{S}I_{S}\left(t\right)-\eta_{S}I_{S}\left(t\right)-\kappa I_{S}\left(t\right)\\ {\dot{I}}_{D}\left(t\right)=\eta_{A}I_{A}\left(t\right)+\eta_{S}I_{S}\left(t\right)-\theta_{D}I_{D}\left(t\right)-\kappa_{D}I_{D}\left(t\right)\\ \dot{R}\left(t\right)=\zeta I_{A}\left(t\right)+\theta_{S}I_{S}\left(t\right)+\theta_{D}I_{D}\left(t\right)\\ \dot{D}\left(t\right)=\kappa I_{S}\left(t\right)+\kappa_{D}I_{D}\left(t\right) \end{array}\right. \end{array}$$ (1)

where *α*, *β*, *γ* are respectively the asymptomatic, symptomatic, and diagnosed transmission rate (i.e. the rate at which asymptomatic, symptomatic, detected individuals cause secondary cases in a population of S susceptible); *δ* is the inverse mean latent period and *ϵ* the asymptomatic percentage; *η*_*A*_, *η*_*S*_ are respectively the asymptomatic and symptomatic detection rates; *ζ*, *θ*_*S*_, *θ*_*D*_ are respectively the asymptomatic, symptomatic, diagnosed healing rates; *κ*, *κ*_*D*_ are respectively the symptomatic and diagnosed mortality rates. The system is closed and positive: all the state variables take non negative values for *t* ≥ 0 if initialized at time 0 with non negative values and satisfy the mass conservation law: $\dot{S}+\dot{E}+{\dot{I}}_{A}+{\dot{I}}_{S}+{\dot{I}}_{D}+\dot{R}+\dot{D}=0$, hence the sum of the states (the total population) is constant.

The formal expression for the basic reproduction number $R_{0}$ in terms of the model parameters can be easily obtained by applying the linearization of the infected subsystem (*E*, *I*_*A*_, *I*_*S*_, *I*_*D*_) of nonlinear ODEs in Eq (1) about the infection-free steady state and evaluating the spectral radius of the corresponding next generation matrix [28], as shown in Appendix A3 4, obtaining

$$\begin{array}{c} R_{0}=\frac{\alpha \epsilon }{\zeta +\eta_{A}}+\frac{\beta (1-\epsilon )}{\theta_{S}+\eta_{S}+\kappa }+\frac{\gamma }{\theta_{D}+\kappa_{D}}[\frac{\epsilon }{\zeta +\eta_{A}}\eta_{A}+\frac{1-\epsilon }{\theta_{S}+\eta_{S}+\kappa }\eta_{S}]. \end{array}$$ (2)

The previous expression has a clear interpretation [29]: the infection process can be triggered from an asymptomatic, a symptomatic or a diagnosed individual, with probability *α*, *β*, *γ* respectively. An asymptomatic individual spends on average a time 1/*δ* in the exposed compartment before entering in the infective asymptomatic compartment with rate *ϵδ*, where she/he spends on average a time 1/(*ζ*+ *η*_*A*_). By multiplying the previous probabilities, the first term in Eq (2) follows. Analogously the infection from a symptomatic individual, that has spent on average a time 1/*δ* in the exposed compartment before entering in the infective symptomatic compartment with a rate (1 − *ϵ*)*δ*, where she/he spends on average a time 1/(*θ*_*S*_ + *η*_*S*_ + *κ*), occurs with a rate *β*. Finally a diagnosed individual, that spends on average a time 1/(*θ*_*D*_+ *κ*_*D*_) in its compartment and can infect with rate *γ*, can either be originated by an asymptomatic individual with rate *η*_*A*_ or by an asymptomatic individual with rate *η*_*S*_, from which the last term with two distinct contributions in Eq (2) follows.

We factorize the transmission rates *α* and *β* in Eq (1) in a pure contact term (i.e. a term that contains information on the contact rate among individuals) and a susceptibility term, *σ*, which takes in account the probability that a potentially contagious contact between a susceptible and an infected individual leads to a new infection:

$$\begin{array}{c} \left\{ \begin{array}{c} \alpha =\alpha_{\text{cont}}\times \sigma \\ \beta =\beta_{\text{cont}}\times \sigma \end{array}\right. \end{array}$$ (3)

where *α*_cont_, *β*_cont_ are the pure contact rate parameters, describing the probability per unit time that a susceptible individual meets an asymptomatic, symptomatic individual, respectively, and depend on the mobility of individuals. Thus we introduce a mobility function *m* and assume the contact rate parameters to have a logarithmic dependence on it:

$$\begin{array}{c} \left\{ \begin{array}{c} \alpha_{\text{cont}}=\alpha_{\text{min}}+\left(\alpha_{\text{max}}-\alpha_{\text{min}}\right)\times \frac{\ln m}{\text{max}(\ln m)}\\ \beta_{\text{cont}}=\beta_{\text{min}}+\left(\beta_{\text{max}}-\beta_{\text{min}}\right)\times \frac{\ln m}{\text{max}(\ln m)} \end{array}\right. \end{array}$$ (4)

where *α*_min,max_ and *β*_min,max_ are appropriate fitting parameters. It is reasonable to expect the symptomatic contact rate parameters to be lower than the corresponding asymptomatic parameters because, even at the beginning of the outbreak, the population was already alerted to avoid contacts with individuals manifesting influenza like symptoms.

One of the most crucial issue in the compartment models is to fix the parameters appearing in Eq (1) and, consequentially also in Eqs (3) and (4)). During an outbreak, the epidemic parameters change in time as a consequence of containment strategies, behavioral changes, modification in the expertise and efficiency of the health care system, modification in the virus stability, etc. In building a model for the epidemic spreading, it is crucial to understand the way in which parameters change. In our model we assume the main epidemiological parameters to be time dependent and try to find reasonable functional behaviors to describe their evolution. The choices made for each parameter are discussed in Appendix A1 4.

## 2 Results

**Fig 2:**
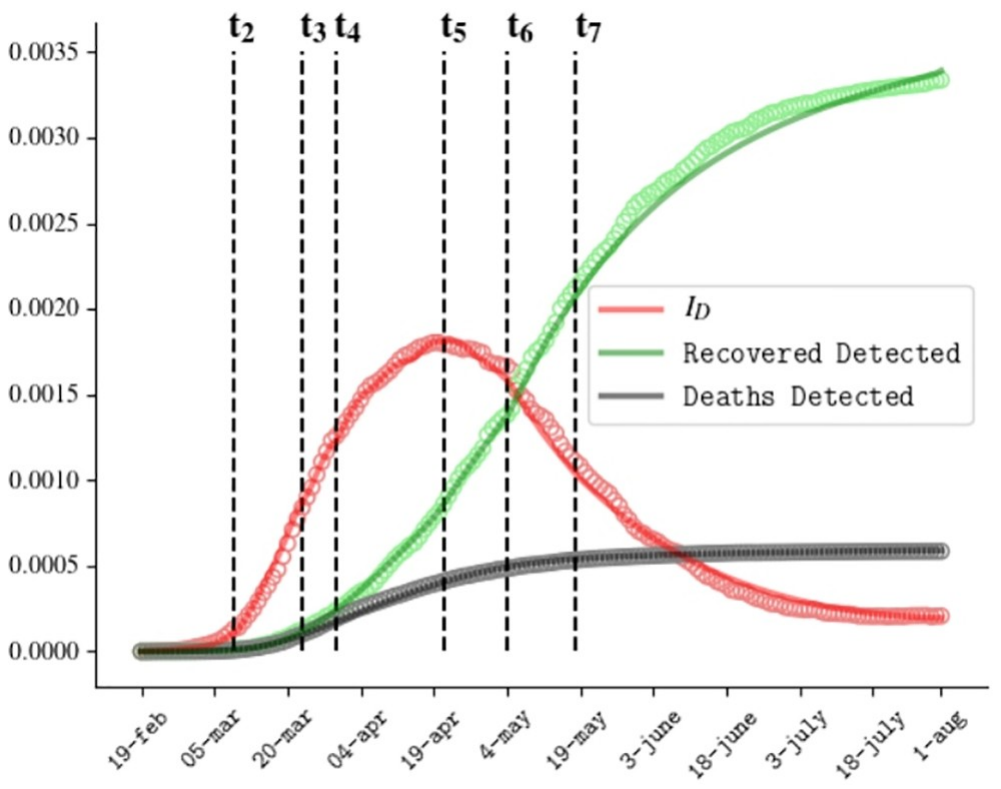
Comparison between numerical resolution (curves) and official data (circles) for the Covid outbreak in Italy for the fraction of active detected cases, recovered detected cases and deaths.

The ODE Eq (1) have been solved using the SciPy libraries. Fig 2 shows the comparison between the predicted evolution of active detected cases, recovered detected cases and dead detected cases in our model, with the official data of the Italian outbreak reported daily by the Health Ministry, from 20th February 2020 to 1st August 2020.

**Fig 3:**
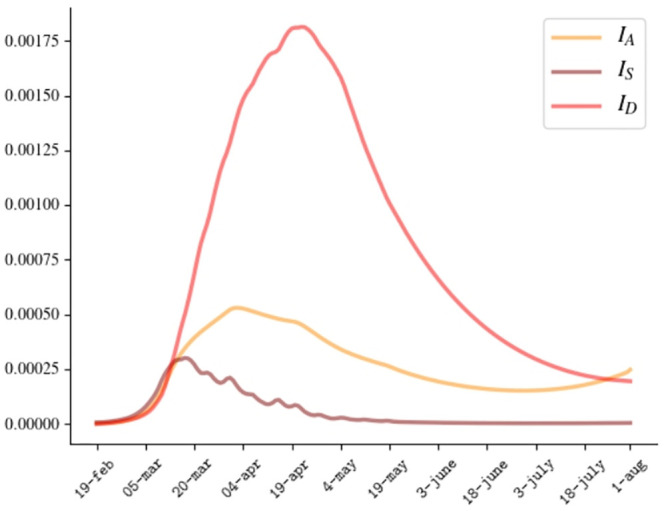
Franction of active cases *I*_*A*_, *I*_*S*_, *I*_*D*_.

Besides the detected cases, our model predicts a relevant number of undetected symptomatic and asymptomatic cases, as shown in Fig 3. According to our prevision, the percentage of undetected cases evolves from 73% at the beginning of March to 55% at the end of July, with a minimum value of 19% at the beginning of May when lockdown ended.

In our model the susceptibility is forged by the risk perception and is expressed as an inverse proportional function of the number of active positive detected cases (see Eq (5)): the higher is the number of diagnosed cases, more scrupulous becomes the adoption of self-protective measures by the population. On the contrary, the reduction of new daily cases induces people to a less rigorous observation of preventive measures. However, we find that, in the very rapid descendant phase of the epidemic beyond the peak (i.e. the time, at which the maximum value of the actual positive cases curve is reached), the agreement between simulated and experimental data can only be obtained by assuming that a further mechanism of susceptibility reduction occurred at the beginning of Phase 2 (i.e. since 4th of May). Such a reduction may be attributed to a potential decline of the stability of the virus in warm environment [30]. Many studies, indeed, explore the effect of temperature and humidity on the number of new cases [31–33] showing that the virulence of the epidemic may be partially reduced with temperature and humidity increases [30].

**Fig 4:**
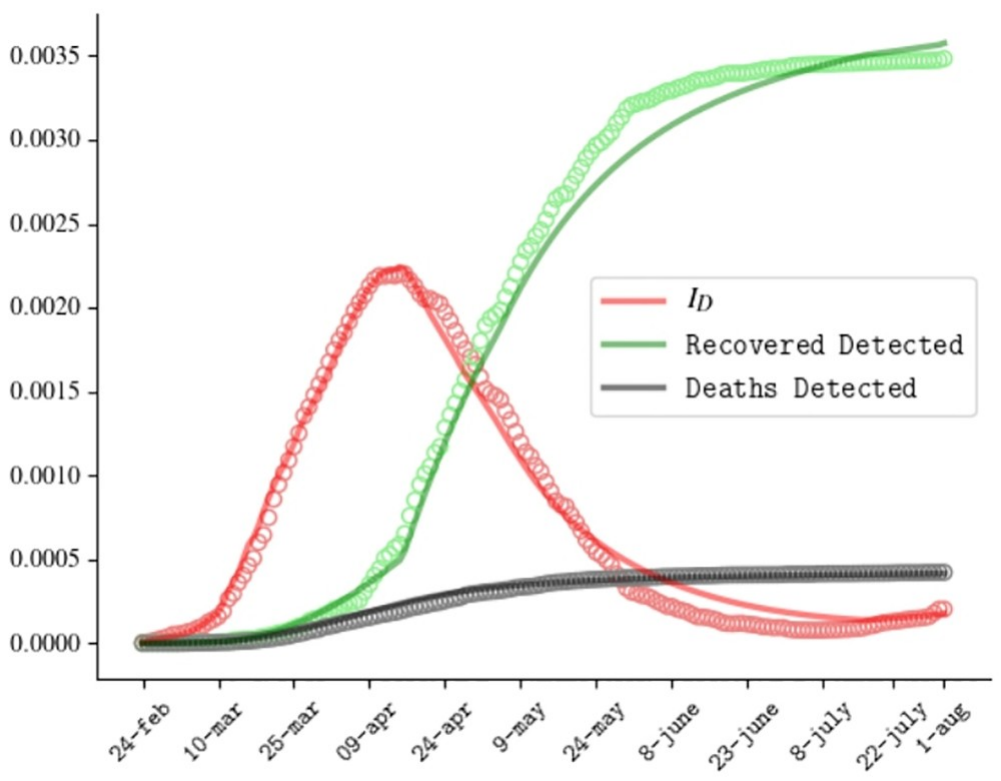
Comparison between numerical resolution (curves) and official data (circles) for the Covid outbreak in Veneto for the active detected cases, recovered detected cases and deaths.

**Fig 5:**
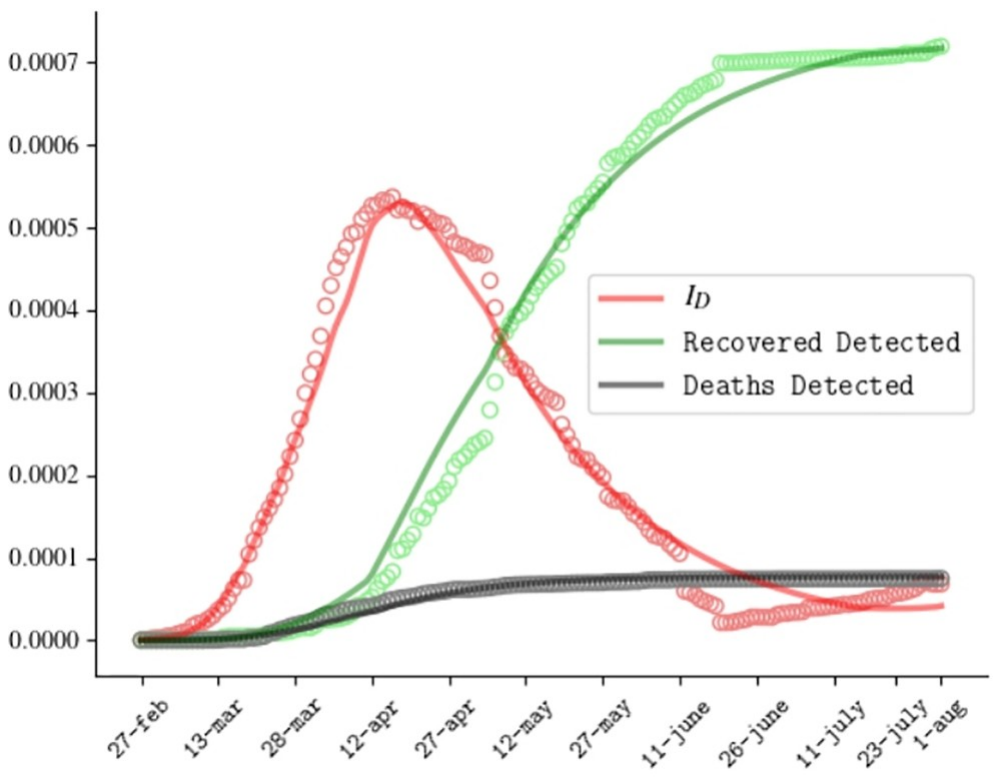
Comparison between numerical resolution (curves) and official data (circles) for the Covid outbreak in Campania region for the active detected cases, recovered detected cases and deaths.

Since the outbreak has spread with different magnitudes in northern versus central/southern regions, in order to further test the model, we also repeat the analysis at the regional level for two sample regions, in which the epidemic had very different size during the first wave, i.e. Veneto (in the North) and Campania (in the South). The comparison between the predicted evolution of the epidemic and the experimental data in the two sample regions are reported in Figs 4 and 5. We find that the model adequately reproduces the epidemic outbreak in the two regions with slight modifications of some fitting parameters, as discussed in Appendix A2 4.

**Fig 6:**
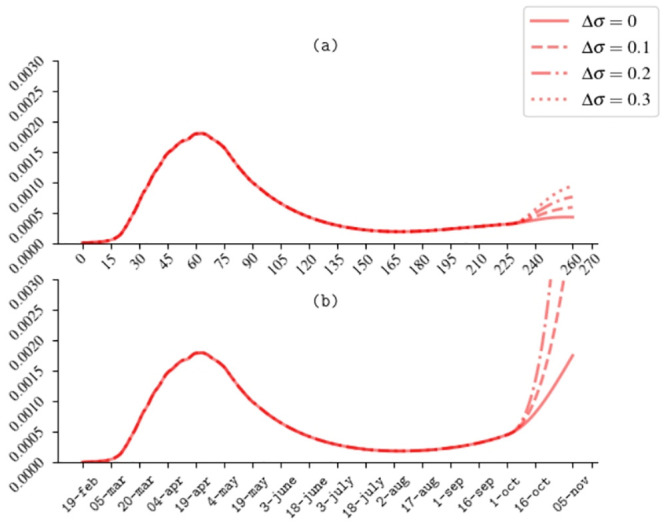
(a) Scenario I with augmented susceptibility in Autumn and awareness mechanism at work; (b) Scenario II with augmented susceptibility in Autumn and without awareness mechanism.

One may wonder if the decrease of temperature in Autumn could increase the number of new daily cases. To explore this scenario, we simulate the effect of a continuous exponential increase in the susceptibility reported in Eq (6), still keeping the inverse proportionality relation with the number of active positive detected cases, since the 1st of October. In Fig 6(a) different scenarios with full mobility restored and different values of Δ*σ*, which represents the maximum value of the susceptibility that can be reached with the awareness mechanism at work, are reported. In absence of any increase in the susceptibility, Δ*σ* = 0, as far as the system may be considered closed, and thus protected from the injection of new cases from abroad, the projections of our model foresee a contained development of the disease with a long tail and with an almost constant number of active cases (around the 0.02% of the total population). So, in presence of an awareness mechanism of risk reduction, a hypothetical increase of the susceptibility, due to climatic factors, would lead to a second wave with an increased fraction of the active cases. However, if the prevalence based mechanism still persists (as stated in Eq (7)), an initially increased number of daily detected cases would raise the risk perception and induce people to a rigorous respect of the preventive hygienic measures. Thus, we find that, even in this case, the epidemic curve would reach a modest peak and then decrease with a long tail. Interestingly we find that the maximum number of active detected cases linearly grows with Δ*σ* according to the law, $I_{D}^{max}=a+b\cdot \Delta \sigma$, with *a* = (1.707±0.001) × 10^−4^ and *b* = (6.573 ± 0.005) × 10^−4^. The duration of the long tail can be significantly reduced, increasing the detection rate of symptomatic and asymptomatic individuals *η*_*A*,*S*_ i.e. performing carpet testing and contact-tracing campaigns.

On the contrary, if we completely turn off the awareness mechanism, at the beginning of August, the epidemic scenario gets definitely worse as shown in Fig 6(b) Thus we conclude that a careful compliance with self-preventive measures is a key factor in containing the intensity of the second wave.

Let us focus on our prevision for the basic reproduction number. As discussed in Section 1 and in Appendix A3 4, this fundamental parameter may be expressed as the sum of three terms: the first one representing the propagation of the epidemic through asymptomatic individuals, the second is the contribution of symptomatic individuals, the third one is the contribution of diagnosed individuals. Their values come from the interplay of all the parameters appearing in the model.

**Fig 7:**
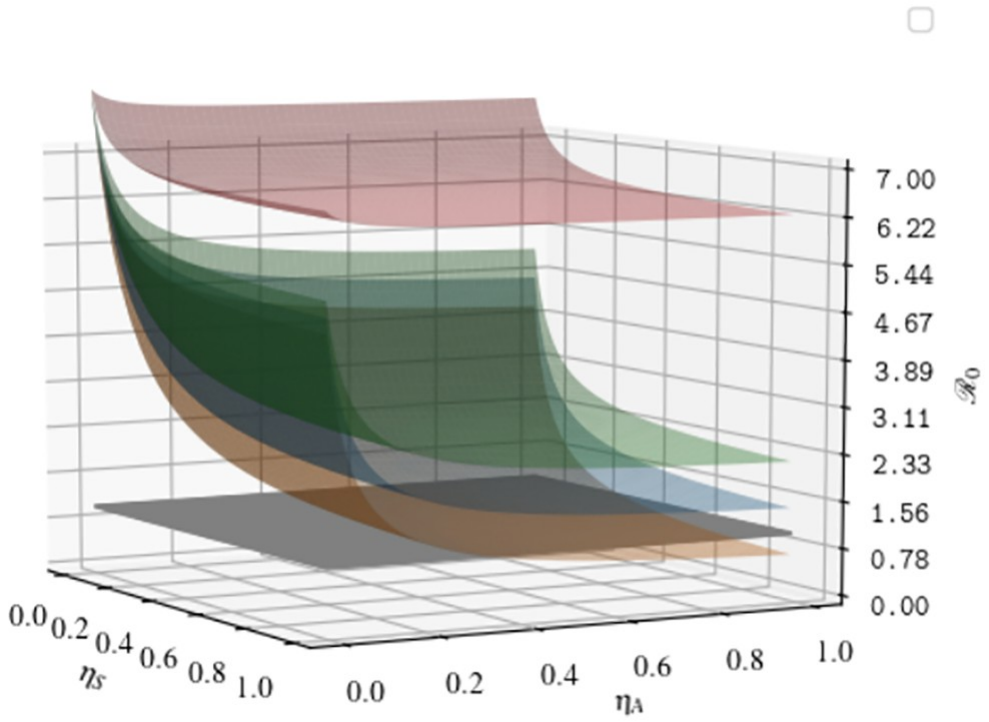
Functional dependence of the basic reproduction number from the detection rates *η*_*A*_, *η*_*S*_ and the transmission rate of the diagnosed compartment *γ*. Black surface: $R_{0}=1$; Orange surface: *γ* = 0.01, Blue surface: *γ* = 0.05, Green surface: *γ* = 0.09, Red surface: *γ* = 0.3. All the remaining parameters are fixed to their value at *t* = 0.

**Fig 8:**
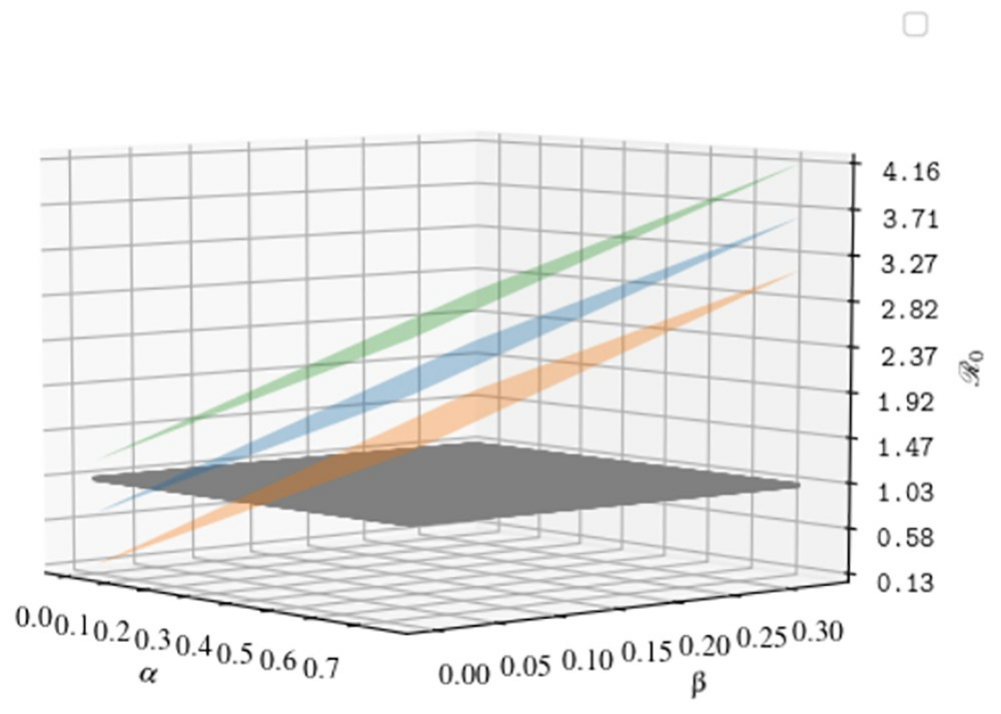
Functional dependence of the basic reproduction number from the transmission rates of undetected individuals (*α* and *β*) and the transmission rate of diagnosed individuals *γ*. Black surface: $R_{0}=1$; Orange surface: *γ* = 0.01, Blue surface: *γ* = 0.05, Green surface: *γ* = 0.09. All the remaining parameters are fixed to their value at *t* = 0.

In Figs 7 and 8 we plot the dependence of $R_{0}$ from the parameters that are not intrinsically dependent on the virus, but can be modified with appropriate containment strategies (i.e. *α*, *β*, *γ*, *η*_*A*_, *η*_*S*_). All the parameters not appearing in the plots are fixed to their initial value at *t* = 0, as reported in Appendix A1 4.

All figures manifest the central role played by the parameter *γ*. If the transmission rate from detected individuals is sufficiently small (i.e. *γ* ≃ 0.01), the value of $R_{0}$ decreases below 1 with increasing the testing efficiency (see Fig 7). On the other hand, if the ability to deal detected individuals with adequate protection, ensuring high level of sanitary isolation, is not sufficiently high, the transmission rate increases in such a way that, no matter how efficient is the testing campaign, the value of $R_{0}$ becomes very large and almost insensitive to the effort in testing (see the surface with *γ* = 0.3 for instance).

**Fig 9:**
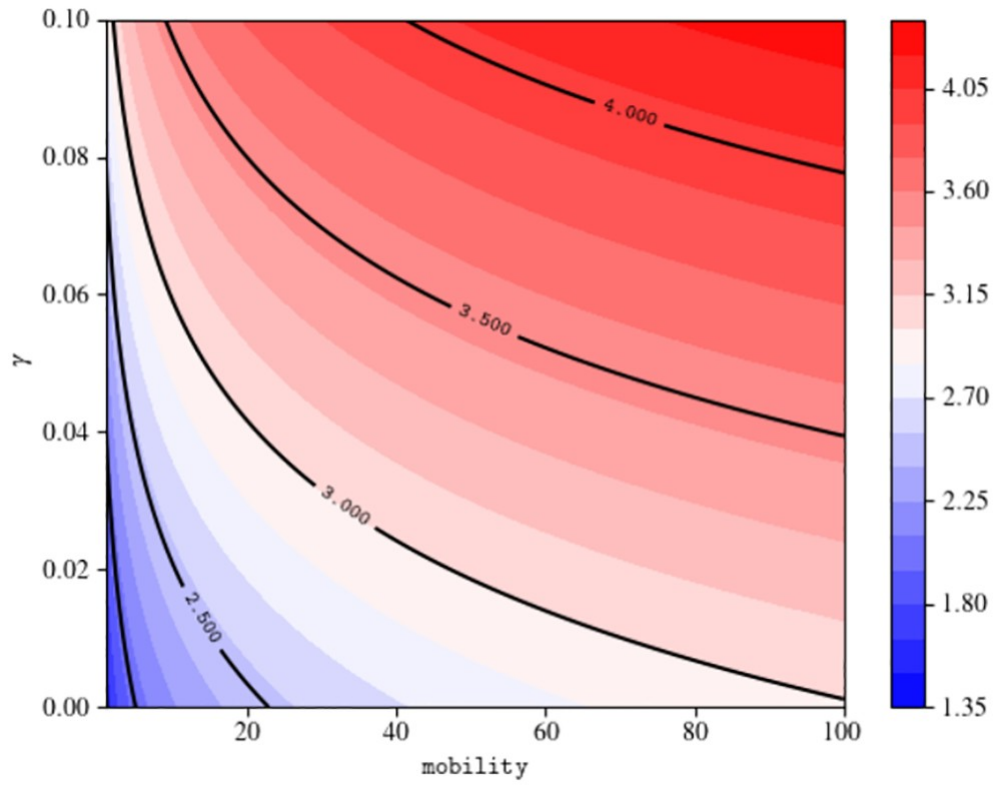
Functional dependence of the basic reproduction number from the mobility and the transmission rate of diagnosed individuals *γ*. All the remaining parameters are fixed to their value at *t* = 0.

Analogously, the behavior of $R_{0}$ as a function of the transmission parameters of the undetected compartments (see Fig 8) reveals that their reduction leads the value of $R_{0}$ below the epidemic threshold only if combined with an adequate isolation of detected cases (*γ* = 0.01, 0.05). One may wonder if the reduction of the mobility parameter *m* within *α*_cont_ and *β*_cont_, without reducing the susceptibility *σ*, is enough to reduce $R_{0}$ below 1. Interestingly, if one explore the dependence of the parameter $R_{0}$ as a function of the mobility *m*, here expressed as a percentage of the full mobility before the outbreak, one can easily see (Fig 9) that, even in an un-realistic scenario with a complete suppression of mobility and very low values for *γ*, the basic reproduction number would stay above the threshold. This circumstance reveals the importance of the parameter *σ* within the transmission rates. We thus find that the adoption of preventive measures, such as wearing masks and gloves, washing hands frequently and all the other self-protective behaviors that may reduce the susceptibility, represented in our model by the parameter *σ*, is crucial to contain the epidemic.

## 3 Discussion

As shown in Appendix A1 1, one of features that distinguishes our *SEI*_*A*_ *I*_*S*_ *I*_*D*_ *RD* model with respect to other similar models in literature is the effort to implement reasonable time dependent parameters, with the aim of reproducing the entire evolution of the epidemic. In Table 1 we report the approach adopted by some papers with respect to the parameters variability. Most of the models make use of step-functions to describe the time evolution of the parameters. We believe this approach to be well suited whenever the parameters under consideration describe punctual events, as for instance lockdown or other containment measures, protocol modifications, reporting modifications, etc. imposed by authorities. The use of step functions should be definitely avoided for all the parameters related to continuous modifications of the epidemic spreading. We tried to minimize the use of step functions in favor of continuous functions, and to relate the few residual discontinuous parameters to specific events. So for instance, the parameter *η*_*A*_, and partially also the healing rates *θ*_*S*,*D*_, have discontinuous behaviors as a consequence of modifications of the testing approach decided by the authorities and occurred in specific days. The other parameter that have an initial discontinuity is the transmission rate from detected individuals *γ*, which may be attributable to the applications of new treatments protocols, or an increased compliance with the sanitary rules to protect sanitary staff and to avoid the epidemic spreading within hospitals and extended care units for elderly people. It was indeed at the end of March (corresponding to the fitting time *t*_4_ in Table 2) that the news about a unusually high number of death in the RSAs (Residenze Sanitarie Assistenziali, i.e. extended care units for elderly people) kept the attention of the media first [36], and of the Italian Health Ministry immediately after [37].

**Table 1:** Models with time dependent parameters.

Paper	Time Evolution
Giordano et al. [25]	step functions
Distante et al. [13]	contact rates: logistic functions
Wangping et al. [23]	transmission rate: step functions
Fanelli et al. [12]	infection rate: exponential function
Gatto et al [26]	transmission rate: step functions
Ivorra et al. [19]	exponential/step functions
Barmparis et al. [21]	contact rate: linear drop
Wang et al. [34]	1 step function
Lalwani et al. [35]	contact rate: step function
Lopez et al. [24]	recovery/death rates: exponential function

**Table 2:** Temporal parameters of the outbreak in Italy and corresponding temporal parameters in the model.

Day	Event	Parameter
20 February	First autochthonous case in Codogno	*t*_0_
26 February	Testing restricted to symptomatic individuals	*t*_1_
9 March	Lockdown—DPCM *“Io Resto a Casa”* [6]	*t*_2_
23 March	Additional restrictive measures—DPCM *“Chiudi Italia”* [41]	*t*_3_
30 March	Fitting parameter	*t*_4_
19 April	Epidemic Peak	*t*_5_
4 May	First DPCM on Phase 2 [40]	*t*_6_
18 May	Second DPCM on Phase 2 [42]	*t*_7_

Another important aspect related to the time evolution of the model parameters is the possibility to infer the time evolution of the basic reproduction number. As well known, such parameter is defined as the average number of secondary infections caused by an infected individual, in a fully susceptible population. For autonomous systems it can be evaluated adopting many different procedures, among which the next generation matrix method, as done for instance in [26], and illustrated for the present case in Appendix A3 4, or similarly recasting the system in a feedback structure and evaluating the static gain, as done in [25]. In the last paper the analytic expression of $R_{0}$ as a function of the time-varying parameters is used to calculate the evolution of the basic reproduction number (that should be better called “effective reproduction number”) across different phases of the epidemic, in which parameters evolve as step-functions. This extension is a delicate issue: time dependent parameters render the system non autonomous. Rigorously speaking, the definition and consequently the evaluation procedure of $R_{0}$ intrinsically make use of the hypothesis that the population is fully susceptible (i.e. *S* = 1 in our notation). Furthermore, in a time-varying environment, the effective reproduction number changes in times both because of the decline of the susceptible compartment and for the modification of some parameters, as a consequence of the implementation of control measures, reduction of the virus stability, improvement in the efficiency of the health care system, and so on. One could observe that, besides some degree of heterogeneity on the national territory, the percentage of infections in Italy during the entire outbreak sum up to a low percentage, estimated to 2.5% from the previously cited serological investigation [27], thus one could still neglect the decline of the susceptible compartment in a short time window. However, discontinuous modifications of the epidemic parameters are not compatible with the use of the analytic expression of $R_{0}$ to infer the value of this parameter during the evolution of the epidemic. One should better derive the effective reproduction number, as done for instance in [38]. We leave this analysis for future work.

Another relevant aspect, emerged from the analysis of the time evolution of the parameters, is the necessity to enforce a reduction of susceptibility in order to catch the very fast decrease in the active cases beyond the peak. We notice that similar hypothesis should be considered also in other models in order to reproduce the experimental data. For instance, considering again the model in [25], and letting their model to evolve from the peak with a significant improvement of testing and contact tracing policies (*ϵ* and *θ* to the maximum value considered in their sensitivity analysis, both leading to a fast suppression of cases), the epidemic curve still has a very long tail with respect to the experimental data. Their solutions get closer to the official data, by considering a strong reduction of the transmission parameters occurring in the same period of our model (at the beginning of May).

It is worth remarking some limitations of the present work. Firstly, in our model the system was assumed to be closed, and thus protected from the injection of new cases from abroad. Such an assumption is perfectly justified up to 3 June, when the frontiers were reopened, and inter-regional mobility was allowed. At the time of revision of the present paper, we know that tourist flows from and to abroad strongly decreased during the Summer 2020, with respect to the previous years, as a consequence of the pandemic, but the inter-regional mobility, specially in August, was intensive. The model should be upgraded in order to include such fluxes. We leave the open system extension of the model to future work.

A second limitation consists in the fact that the model cannot distinguish the symptomatic cases according to the severity of symptoms, since pauci, mild and severe symptomatic cases all together flow in the symptomatic compartment. Analogously the number of hospitalized, intensive care and home isolated cases cannot be obtained by our model, since all diagnosed individuals flow in the *I*_*D*_ compartment. It is possible to rearrange the model in order to capture these aspects, but this would lead to an increase in the number of state variables and model parameters.

Finally the model does not take into account the age structure of population, therefore the results obtained are not able to explain, for example, differences in infections, mortality and recovering among different age classes, observed by the health surveillance data. Again the introductions of new age compartments, and relative parameters, can be implemented in order to include the age structure in the model.

## 4 Conclusion

In this paper we developed a deterministic compartment model to study the entire evolution of the Covid-19 outbreak in Italy. We made a retrospective analysis to understand what kind of modifications occurred to the characteristic parameters that regulate the epidemic spreading, as a consequence of containment strategies, behavioral changes, modification in the expertise and efficiency of the health care system, modification in the virus stability, and so on. We made the effort to implement reasonable time dependent functions and reproduce the entire evolution of the epidemic with very satisfactory agreement with experimental data. According to our model a reduction of susceptibility, possibly due a reduction of the virus stability with high temperature, should have been occurred in order to justify the very fast decrease in the number of active cases beyond the peak.

From the analysis of the behavior of the basic reproduction number in response to variation of those epidemiological parameters that can be influenced by the policy makers, we learn that one of the most relevant aspect for containing the outbreak is a significant reduction of the transmission rate from diagnosed cases. Thus we believe that a rigid isolation procedure for the detected cases, combined with an intensive effort in extended testing campaigns, is of primary importance to fight against Covid-19 pandemic.

We also proved that, as far as the system may be considered closed, and thus protected from the injection of new cases from abroad, strict compliance of self-protective measures is crucial in order to contain the second wave in a fully restored mobility scenario with partially increased susceptibility.

## Appendix

### A1. The time evolution of parameters

In this section we discuss the choices made for the time evolution of each parameter appearing in Eqs (1), (3) and (4). We should stress that the procedure of fitting 12 parameters, with their time variability, does not allow to univocally identify them. This is true in most of the compartmental models published in literature, with more than 2 − 3 compartments. The idea is to exhibit a possible set of parameters, that, within the specific model/formulation, allows to reproduce the epidemic evolution. The parameters are intended as regulators of fluxes between the compartments of a specific model.

**The susceptibility *σ***—For what concerns the risk perception, we assume a prevalence based mechanism of rising awareness, i.e. the time evolution of the awareness is functionally dependent on the time evolution of the number of infective detected individuals, without any temporary effect of amplification or falsification [39].

In particular, starting with the first lockdown measure imposed by the Italian government on *t*_2_ = 9 March, at each time t, we assume the susceptibility to be inversely proportional to the number of active detected cases *I*_*D*_. On this awareness conditioned susceptibility reduction, we add with continuity a further compression effect since the beginning of Phase 2 (*t*_6_ = 4 May), when a first reduction of the mobility restrictions was decided by the Italian Government [40]

$$\begin{array}{c} \sigma \left(t\right)=\{\begin{array}{c} \frac{I_{D}\left(t_{2}\right)}{I_{D}\left(t\right)}\;\;\;\text{for}\;\;\;t_{2}\leq t\leq t_{6}\\ \sigma_{1}\frac{I_{D}\left(t_{2}\right)}{I_{D}\left(t\right)}+\left(1-\sigma_{1}\right)\frac{I_{D}\left(t_{2}\right)}{I_{D}\left(t_{6}\right)}\;\;\;\text{for}\;\;\;t\geq t_{6} \end{array} \end{array}$$ (5)

where *σ*_1_ is a fitting parameter. The main temporal parameters of the Italian outbreak, relevant for the present model, are reported in Table 2.

As discussed in the Discussion section, this further mechanism of susceptibility reduction seems to be necessary in order to explain the descendant phase of the epidemic curve in Italy.

For the epidemic spreading in the cold months, we consider two possible scenarios. In the first one, the evolution of the susceptibility, *σ*(*t*), since the beginning of October is assumed to be:

$$\begin{array}{c} \sigma \left(t\right)=\sigma \left(t^{*}\right)\frac{I_{D}\left(t^{*}\right)}{I_{D}\left(t\right)}+\Delta \sigma (1-e^{-k_{\sigma }\left(t-t^{*}\right)})\frac{I_{D}\left(t^{*}\right)}{I_{D}\left(t\right)}\;\;\;\text{with}\;\;\;t^{*}=1\text{st}\;\;\text{October} \end{array}$$ (6)

The parameter Δ*σ* determines the maximum value of the susceptibility that can be reached with the awareness mechanism at work, while the parameter *k*_*σ*_ fixes the speed at which the susceptibility increases.

In the second scenario, the awareness mechanism is frozen since the beginning of August and the evolution in the cold months is governed by the following expression for *σ*(*t*):

$$\begin{array}{c} \sigma \left(t\right)=\sigma \left(t^{*}\right)+\Delta \sigma \left(1-e^{-k_{\sigma }\left(t-t^{*}\right)}\right)\;\;\;\text{with}\;\;\;t^{*}=1\text{st}\;\;\text{October}. \end{array}$$ (7)

**The mobility function *m***—This parameter is shaped by the social distancing measures, and in particular by the lockdown measures. In Italy, a strong reduction of the mobility has been registered starting with 9th March, as a consequence of the lockdown decided by the Italian Government on the whole national territory [6]. The Google Covid-19 Community Mobility Report [43] furnishes information on the modification occurred on the community movements in specific locations (retail and recreation, grocery and pharmacy, parks, transport, workplaces, residential) since the beginning of the outbreak. From those data we construct a mobility function *m*(*t*) as the weighted average on the mobility data on different places. In order to fix the weights, we assume that the risk of a local outbreak is strictly related to crowding and duration of interaction, as shown by [44] who studied the epidemiology of a Covid outbreak in a call center in South Corea. In this research authors found that that the attack rate of the virus was 8.5% among co-workers in the same building, but it increased up to 43.5%, when limiting the analysis to a specific floor. In other words high-density environments are high-risk sites, and the duration of interaction seems to be the main facilitator for the spreading of Covid-19. Thus we attribute an exposition factor to each location, obtained as the product of the average density of people in a specific location times the average time spent in each context. The weights used in the mobility function are chosen in such a way to be in the same ratio as the exposition factors of different locations. The original mobility data set and the emerging mobility function are reported in S1 Fig in S1 File.

**The transmission rate *γ***—Diagnosed individuals are subject to quarantine and eventually recovered in hospitals. Thus the possibility for a susceptible individual to get in contact with a diagnosed one reduces to the household or the hospital context and it is not affected by the mobility reduction previously discussed. On the other hand, the efficiency in treating infected individuals with the appropriate protection protocols increased during the pandemic. For this reason we assume that the transmission rate *γ* changed in time with respect to the first phase of the epidemic. In particular we assume *γ* to increase discontinuously up to the peak (*t*_5_ = 19 April) and then to decrease exponentially as follows

$$\begin{array}{c} \gamma \left(t\right)=\{\begin{array}{c} \gamma_{1}\;\;\;\text{for}\;\;\;t\leq t_{4}\\ \gamma_{2}\;\;\;\text{for}\;\;\;t_{4}\leq t\leq t_{5}\;\;\;\text{with}\;\;\;\gamma_{2}\leq \gamma_{1}\\ \gamma_{3}e^{-\gamma_{4}\left(t-t_{5}\right)}\;\;\;\text{for}\;\;\;t\geq t_{5}\;\;\;\text{with}\;\;\;\gamma_{3}\leq \gamma_{2} \end{array} \end{array}$$ (8)

where *γ*_1−4_ are fitting parameters. The time evolution of the parameters *α*, *β*, *γ* is plotted in S2 Fig in S1 File.

**The fraction of asymptomatic individuals *ϵ***—Estimations for the fraction of asymptomatic individuals in literature vary between 18% [45] and 80% [46], with an estimate of 94% among children [47]. According to the previously cited serological investigation in [27], the percentage of completely asymptomatic individuals is 27%.

In Italy, a reduction of the manifestation of symptoms has been registered with an increased percentage of pauci-symptomatic and asymptomatic individual with the incoming of the hot season. According to the data published by Istituto Superiore di Sanità, the fraction of asymptomatic and pauci-symptomatic infected individuals is increased from 15% [48] in March to 55% in July [49]. Such an increase may be due to the combined effect of more extended testing campaign in the descendant phase of the epidemic and an effective reduction of mild and severe cases. We assume the percentage of asymptomatic cases to be constant up to the peak and, afterwards, to slightly increase according to an exponential law with parameter *ϵ*_*gr*_

$$\begin{array}{c} \epsilon =\{\begin{array}{c} \epsilon_{\text{min}}\;\;\;\text{for}\;\;\;t\leq t_{5}\\ \epsilon_{\text{min}}+\left(\epsilon_{\text{max}}-\epsilon_{\text{min}}\right)\left(1-e^{-\epsilon_{\text{gr}}\left(t-t_{5}\right)}\right)\;\;\;\text{for}\;\;\;t\geq t_{5} \end{array} \end{array}$$ (9)

with ∊_max,min,gr_ fitting parameters. The time evolution of the asymptomatic fraction is plotted in S3 Fig in S1 File.

**The detection rates *η*_*A*,*S*_**—At the very beginning of the outbreak, as soon as an infected individual was detected, all the persons that had contacts with the infected case, were tested against Covid-19. However, since *t*_1_ = 26th of February, authorities stated that only individuals with expressed symptoms could have access to testing. This circumstance excluded the possibility to detect asymptomatic infected individuals for a long period. We thus assume *η*_*A*_ to have had a window between *t*_1_ = 26th of February and *t*_7_ = 18th of May (full implementation of Phase 2) during which no asymptomatic cases could be detected

$$\begin{array}{c} \eta_{A}\left(t\right)=\{\begin{array}{c} \eta_{\text{min}}\;\;\;\text{for}\;\;\;t\leq t_{1}\\ 0\;\;\;\text{for}\;\;\;t_{0}\leq t\leq t_{7}\\ \eta_{\text{min}}/10\;\;\;\text{for}\;\;\;t\geq t_{7} \end{array} \end{array}$$ (10)

where *η*_min_ is the minimum value of the symptomatic detection rate. The reduction factor in the detection rate since Phase 2 is motivated by the circumstance that the access to testing continued to be prioritily reserved to symptomatic individuals.

The detection rate of symptomatic individuals changed during the outbreak. In Phase 1, even if the number of swab tests increased significantly, the number of individuals that needed to be tested increased even faster, as shown in the ISS Extended report (see for instance [49, 50]) where the time evolution of the median time interval between the onset of symptoms and the access to testing is reported. We assume that the testing capacity in Phase 1 was saturated and thus we take the detection rate to be proportional to the number of swabs performed daily, in that time window. On the contrary we assume that, since Phase 2, new symptomatic individuals are promptly tested, and thus we keep the detection rate to be constant and equal to the maximum value reached at *t*_7_ (i.e. the full implementation of Phase 2).

$$\begin{array}{c} \eta_{S}\left(t\right)=\{\begin{array}{c} \left(\eta_{\text{max}}-\eta_{\text{min}}\right)\times \frac{\text{test}(t)}{\text{max}(\text{test}(t))}+\eta_{\text{min}}\;\;\;\text{for}\;\;\;t\leq t_{7}\\ \eta_{\text{max}}\;\;\;\;\text{for}\;\;\;t\geq t_{7} \end{array} \end{array}$$ (11)

with *η*_max,min,gr_ fitting parameters. The time evolution of the detection rate parameters is plotted in S4 Fig in S1 File.

**The healing rates *ζ*, *θ*_*S*_, *θ*_*D*_**—We assume a constant healing rate *ζ* for asymptomatic individuals and fix its value to 1/14 day^−1^, corresponding to the an healing time of 14 days [51].

For symptomatic and diagnosed individuals, we assume that the healing rate has improved during the outbreak as a consequence of increased effectiveness of treatments. As previously discussed, from 26th February till the beginning of May, the access to swab tests was limited to symptomatic individuals. Extend testing campaigns started to be realized only at the beginning of Phase 2 in order to admit people at work. We thus assume the healing rate of infected symptomatic and diagnosed to evolve together until Phase 2. Later the fraction of asymptomatic cases within the diagnosed ones increased, thus we expect the healing rate of detected individuals coming closer to the value of *ζ* (i.e. the healing rate for asymptomatic individuals). We assume these rates to evolve as follows

$$\begin{array}{c} \theta {\left(t\right)}_{S,D}=\{\begin{array}{c} \theta_{1}\;\;\;\text{for}\;\;\;t\leq t_{5}\\ \theta_{2}\;\;\;\text{for}\;\;\;t_{5}\leq t\leq t_{6}\;\;\;\text{with}\;\;\;\theta_{1}\leq \theta_{2}\\ \theta_{3S,3D}\;\;\;\text{for}\;\;\;t\geq t_{6}\;\;\;\text{with}\;\;\;\theta_{2}\leq \theta_{3S}\leq \theta_{3D} \end{array} \end{array}$$ (12)

*θ*_1,2,3*S*,3*D*_ are fitting parameters. The time evolution of the detection rate parameter is plotted in in S5 Fig in S1 File. We remember that diagnosed individuals were declared ‘recovered’ after a double negative swap. This definition of detected recovered may lead to an overstimate of the infectious period. However systematic data on the clinical recovery are not available at the moment.

**The mortality rates *κ*, *κ*_*D*_**—For the mortality rate we assume it to follow an exponential decay, as in [52] both for diagnosed and symptomatic cases

$$\begin{array}{c} \kappa \left(t\right)=\kappa_{D}\left(t\right)=\kappa_{0}e^{-\kappa_{1}t} \end{array}$$ (13)

where *κ*_1,2_ are fitting parameters. In order to have a non zero mortality, we used a cutoff value *k*_min_ = 1.4 × 10^−3^. The best fit parameters, obtained minimizing the *χ*-square with respect to the experimental data, for the Italian data are reported in Table 3.

**Table 3:** Fitting parameters, obtained with *E*_0_ = 300/*N*, $I_{A_{0}}=200/N$, $I_{S_{0}}=500/N$, $I_{D_{0}}=3/N$, corresponding to an estimate of 1000 cases in the first week.

*α*_min_	0.38 day^−1^
*α*_max_	0.76 day^−1^
*β*_min_	0.16 day^−1^
*β*_max_	0.33 day^−1^
*γ*_1_	9 × 10^−2^ day^−1^
*γ*_2_	3.8 × 10^−2^ day^−1^
*γ*_3_	1.1 × 10^−2^ day^−1^
*γ*_4_	1.6 × 10^−2^ day^−1^
*δ*	1/3 day^−1^
*∊*_min_	0.35
*∊*_max_	0.9
*∊*_gr_	0.02
*ζ*	1/14 day^−1^
*η*_min_	0.1 day^−1^
*η*_max_	0.9 day^−1^
*θ*_1_	0.017 day^−1^
*θ*_2_	0.023 day^−1^
*θ*_3*S*,3*D*_	0.030, 0.039 day^−1^
*κ*_0_	0.32 day^−1^
*κ*_1_	0.31 day^−1^
*σ*_1_	0.2

### A2. Epidemic spreading in Veneto and Campania

As shown in Figs 4 and 5, the model adequately reproduces the epidemic outbreak in the two regions with slight modifications of some fitting parameters. In particular, we find some differences in contact rates, with higher contact coefficients in Campania, that is the region with the highest population density in Italy, than in Veneto, that has a population density lower than the Italian mean value. Furthermore we find different recovering rates of detected individuals among the two regions, possibly due to differences in waiting times of the test swabs necessary to ascertain the recovering status.

The best fit parameters, for the regional cases obtained minimizing the *χ*-square with respect to the experimental data, for the regional data are reported in Table 4.

**Table 4:** Fitting parameters for Veneto and Campania regions.

Parameter	Veneto	Campania
*α*_min_	0.35 day^−1^	0.4 day^−1^
*α*_max_	0.72 day^−1^	0.81 day^−1^
*β*_min_	0.12 day^−1^	0.2 day^−1^
*β*_max_	026 day^−1^	0.4 day^−1^
*γ*_1_	7 × 10^−2^ day^−1^	11 × 10^−2^ day^−1^
*γ*_2_	1.75 × 10^−2^ day^−1^	7.3 × 10^−2^ day^−1^
*γ*_3_	1.75 × 10^−2^ day^−1^	9.16 × 10^−2^ day^−1^
*γ*_4_	1.20 × 10^−1^ day^−1^	1.60 × 10^−1^ day^−1^
*δ*	1/3 day^−1^	1/3 day^−1^
*∊*_min_	0.35	0.35
*∊*_max_	0.9	0.9
*∊*_gr_	0.02	0.02
*ζ*	1/14 day^−1^	1/14 day^−1^
*η*_min_	0.1 day^−1^	0.1 day^−1^
*η*_max_	0.9 day^−1^	0.9 day^−1^
*θ*_1,*S*_	0.017 day^−1^	0.017 day^−1^
*θ*_2,*S*_	0.023 day^−1^	0.023 day^−1^
*θ*_3,*S*_	0.03 day^−1^	0.03 day^−1^
*θ*_1,*D*_	0.01 day^−1^	0.01 day^−1^
*θ*_2,*D*_	0.04 day^−1^	0.023 day^−1^
*θ*_3,*D*_	0.05 day^−1^	0.034 day^−1^
*κ*_0_	0.032 day^−1^	0.032 day^−1^
*κ*_1_	0.031 day^−1^	0.031 day^−1^
*κ*_0,*D*_	0.011 day^−1^	0.011 day^−1^
*κ*_1,*D*_	0.023 day^−1^	0.026 day^−1^
*κ*_min_	2.3 × 10^−4^ day^−1^	3.74 × 10^−4^ day^−1^
*σ*_1_	0.03	0.1

The regional simulations are initiated at the time of the first entry in the regional infected data (24th February and 27th February respectively in Veneto and Campania) using available data for *I*_*D*_, *R*, *D*. Unobservable initial data (*E*_0_, *I*_*A*__0_, *I*_*S*__0_,) are inferred from those obtained from the simulation at national level.

### A3. Fit of Italian data up to 30th September

In writing the revised version of the paper, we felt it appropriate to test the model on new epidemiological data available up to 30th of September. Some observation are due:

- The holiday period was characterized by a generalized desire to get behind the Covid spectrum, causing many people to relax and significantly reduce the attention to compliance with social distancing, use of masks and general preventive measures.
- At the mid of August a significant increase in the number of test swabs was registered as a consequence of the Italian Health Ministry ordinance [53], which imposed test swabs in airport on people arriving from Malta, Spagna and Grecia, and on their contacts, if positive.

**Fig 10:**
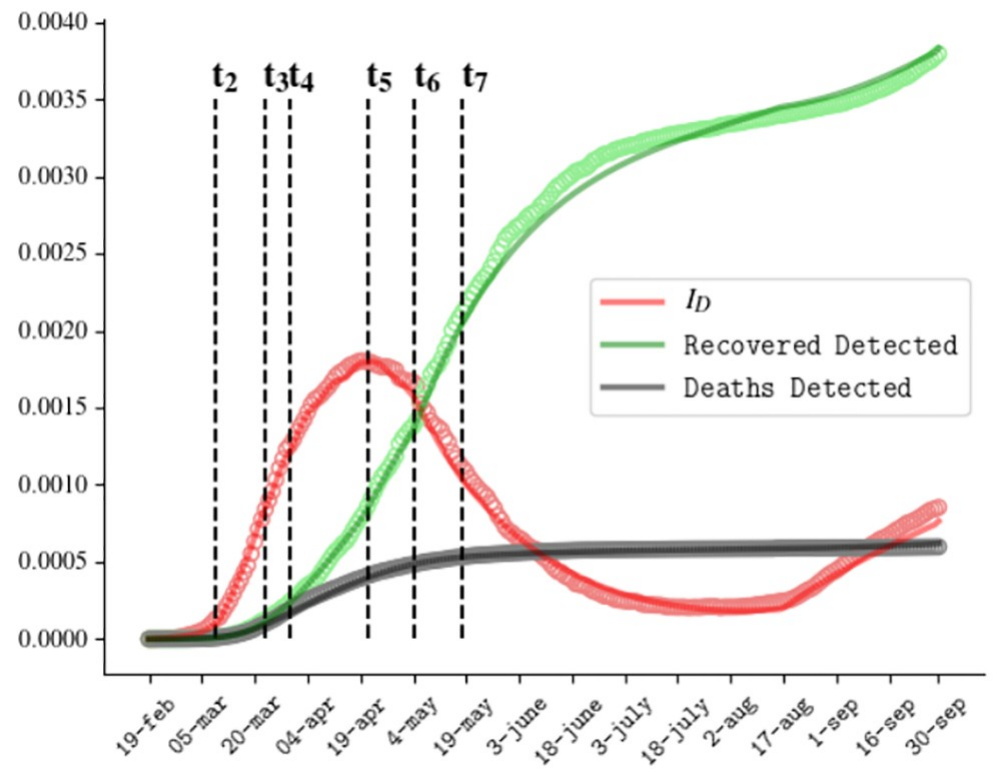
Comparison between numerical resolution (curves) and official data (circles) for the Covid outbreak in Italy up to 30th September for the active detected cases, recovered detected cases and deaths. Awareness mechanism suspended since 1st August; *η*_*A*_ = 0.04*day*^−1^, *θ*_*D*_ = 0.017*day*^−1^ since 14th August.

Consistently with these circumstances, we perform a simulation in which the awareness mechanism of risk reduction is frozen since the 1st of August, the asymptomatic detection rate is increased since the 14th of August and the recovery rate for diagnosed people is slightly decreased, in order to take in account the lengthening of the waiting time for the two consecutive negative swabs attesting recovery, compared to the waiting time for hospitalized individuals. Numerical resolution is reported in Fig 10, in comparison with experimental data. Our findings suggest that the current state of epidemic in Italy is essentially due to the full neglect of risk containment measures during the Summer period.

We do not extend this analysis at regional level, since the possibility to treat the system as a closed universe (that is still reasonable at national level, in consideration of the reduced fluxes of people from and to abroad during the summer) definitely cannot be applied at regional level because the inter-regional mobility was very strong.

### A4. Evaluation of the basic reproduction number R

To evaluate the basic reproduction number, following [28], we consider the infected subsystem **x** = (*E*, *I_A_*, *I_S_*, *I_D_*) of nonlinear ODEs in Eq 1 and linearize it about the infection-free steady state (*E* = 0, *I*_*A*_ = 0, *I*_*S*_ = 0, *I*_*D*_ = 0) with *S* = 1, obtaining

$$\begin{array}{c} \left\{ \begin{array}{c} \dot{E}\left(t\right)=\alpha I_{A}\left(t\right)+\beta I_{S}\left(t\right)+\gamma I_{D}\left(t\right)-\delta E\\ {\dot{I}}_{A}\left(t\right)=\epsilon \delta E\left(t\right)-\zeta I_{A}\left(t\right)-\eta_{A}I_{A}\left(t\right)\\ {\dot{I}}_{S}\left(t\right)=\left(1-\epsilon \right)\delta E\left(t\right)-\theta_{S}I_{S}\left(t\right)-\eta_{S}I_{S}\left(t\right)-\kappa I_{S}\left(t\right)\\ {\dot{I}}_{D}\left(t\right)=\eta_{A}I_{A}\left(t\right)+\eta_{S}I_{S}\left(t\right)-\theta_{D}I_{D}\left(t\right)-\kappa_{D}I_{D}\left(t\right) \end{array}\right. \end{array}$$ (14)

that can be recast in the following form

$$\begin{array}{c} \dot{x}=\left(T+\Sigma \right)x \end{array}$$ (15)

where **T** is the transmission matrix that describes the production of new infections,

$$\begin{array}{c} T=(\begin{array}{cccc} 0 & \alpha & \beta & \gamma \\ 0 & 0 & 0 & 0\\ 0 & 0 & 0 & 0\\ 0 & 0 & 0 & 0 \end{array}) \end{array}$$ (16)

and **Σ** is the transition matrix that take in account the fluxes among compartments

$$\begin{array}{c} \Sigma =(\begin{array}{cccc} -\delta & 0 & 0 & 0\\ \epsilon \delta & -(\zeta +\eta_{A}) & 0 & 0\\ (1-\epsilon )\delta & 0 & -(\theta_{S}+\eta_{S}+\kappa ) & 0\\ 0 & \eta_{A} & \eta_{S} & -(\theta_{D}+\kappa_{D}) \end{array}) \end{array}$$ (17)

Notice that every new infected individual naturally enters in the latency compartment, while an individual that move from the latency to the infective compartments do not correspond to a new infection, but to an infected individual moving to a different infection stage. This is the reason why the matrix **T** has only one raw with entries different from zero.

The Next Generation Matrix with large domain (i.e. with the inclusion of all the infective states and not only the new infections) is thus obtained as

$$\begin{array}{c} K_{L}=-T\Sigma^{-1}=(\begin{array}{cccc} k_{1} & k_{2} & k_{3} & k_{4}\\ 0 & 0 & 0 & 0\\ 0 & 0 & 0 & 0\\ 0 & 0 & 0 & 0 \end{array}) \end{array}$$ (18)

where

$$\begin{array}{c} \left\{ \begin{array}{c} \kappa_{1}=\frac{\epsilon \alpha }{\zeta +\eta_{A}}+\frac{\beta (1-\epsilon )}{\theta_{S}+\eta_{S}+\kappa }+\frac{\gamma }{\theta_{D}+\kappa_{D}}(\frac{\epsilon \eta_{A}}{\zeta +\eta_{A}}+\frac{\left(1-\epsilon \right)\eta_{S}}{\theta_{S}+\eta_{S}+\kappa })\\ \kappa_{2}=\frac{\alpha }{\zeta +\eta_{A}}+\frac{\gamma \eta_{A}}{\left(\zeta +\eta_{A}\right)\left(\theta_{D}+\kappa_{D}\right)}\\ \kappa_{3}=\frac{\beta }{\theta_{S}+\eta_{s}+\kappa }+\frac{\gamma \eta_{s}}{\left(\theta_{S}+\eta_{S}+\kappa \right)\left(\theta_{D}+\kappa_{D}\right)}\\ \kappa_{4}=\frac{\gamma }{\theta_{D}+\kappa_{D}} \end{array}\right. \end{array}$$ (19)

The basic reproduction number is thus obtained as the spectral radius of the previous matrix and can be written as the sum of three contribution, each describing the infection process triggered by one compartment of infective individuals (Asymptomatic, Symptomatic, Diagnosed)

$$\begin{array}{c} R_{0}=R_{0}^{A}+R_{0}^{S}+R_{0}^{D} \end{array}$$ (20)

where

$$\begin{array}{c} R_{0}^{A}=\frac{\epsilon \alpha }{\zeta +\eta_{A}};\;\;\;R_{0}^{S}=\frac{\beta (1-\epsilon )}{\theta_{S}+\eta_{S}+\kappa };\;\;\;R_{0}^{D}=\frac{\gamma }{\theta_{D}+\kappa_{D}}(\frac{\epsilon \eta_{A}}{\zeta +\eta_{A}}+\frac{\left(1-\epsilon \right)\eta_{S}}{\theta_{S}+\eta_{S}+\kappa }) \end{array}$$ (21)

## Supporting information

S1 File (PDF)
