# Supplementary material for: Beyond the peak: A deterministic compartment model for exploring the Covid-19 evolution in Italy
Source: PLoS One. 2020 Nov 6;15(11):e0241951. doi: 10.1371/journal.pone.0241951 (PMC7647079; doi:10.1371/journal.pone.0241951)
Supplement: S1 File — (PDF) [file pone.0241951.s001.pdf]

Supplementary Information

Beyond the peak: a deterministic compartment model for exploring the Covid-19 evolution in Italy

Silvio Romano, Annalisa Fierro, Antonella Liccardo

The present supplementary information contains some figures related to the evolution of parameters.

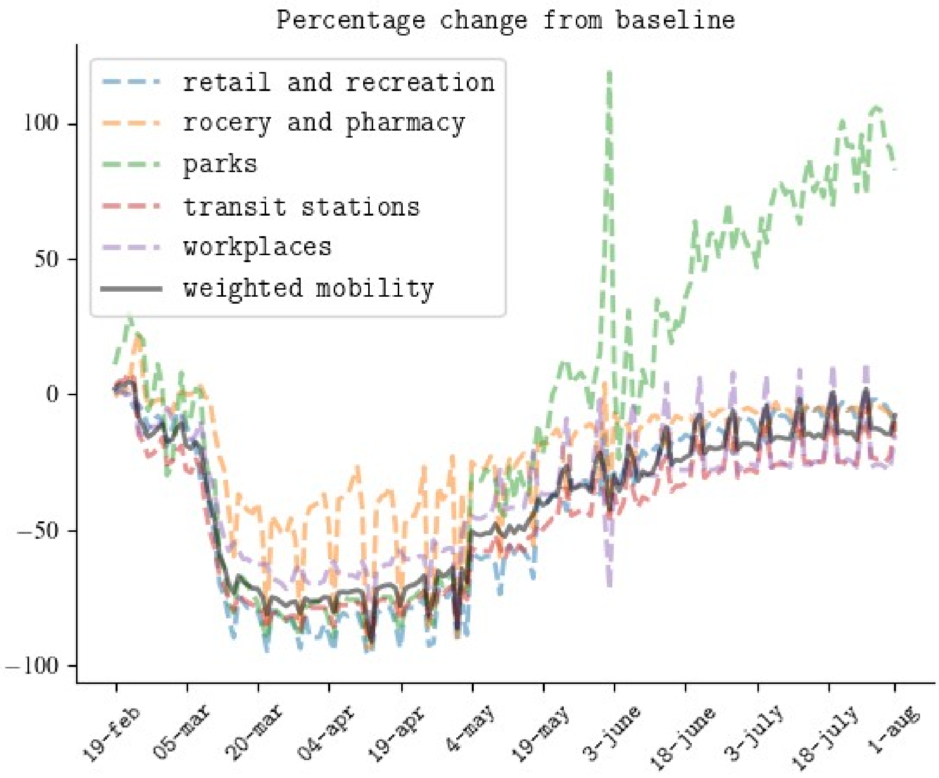

S1 Fig. Weighted mobility function m (t) (black line) extracted from the Google Mobility Reports data (colored lines), with the following weights: retail and recreation (0.2), grocery and pharmacy (0.05), parks (0.05), transport (0.4), workplaces (0.3).

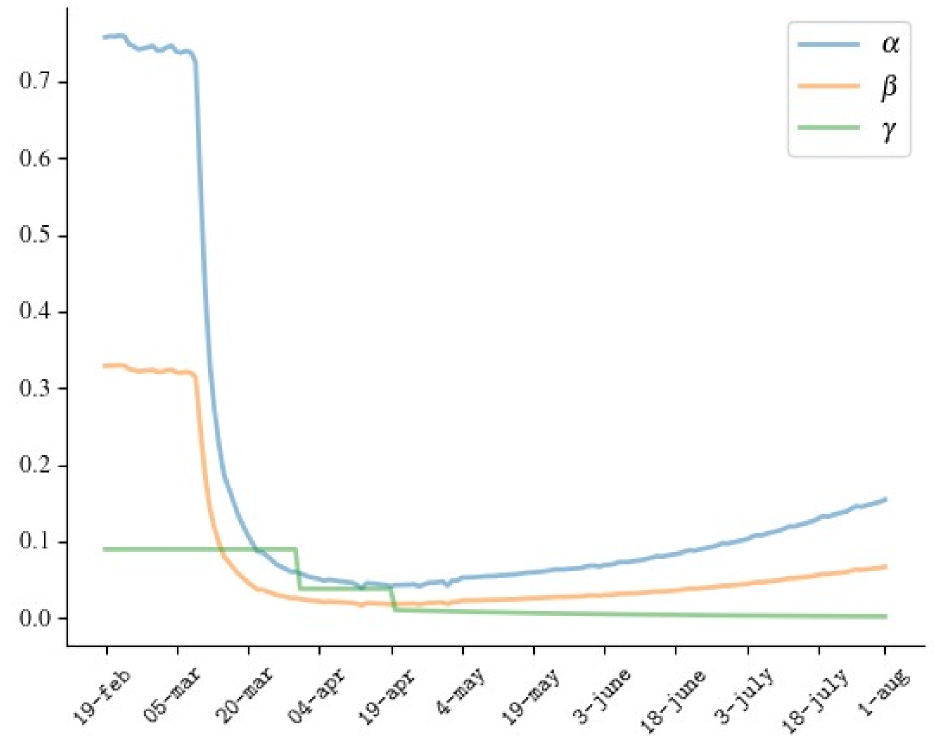

S2 Fig. Time evolution of the transmission parameters α(t), β(t), γ(t).

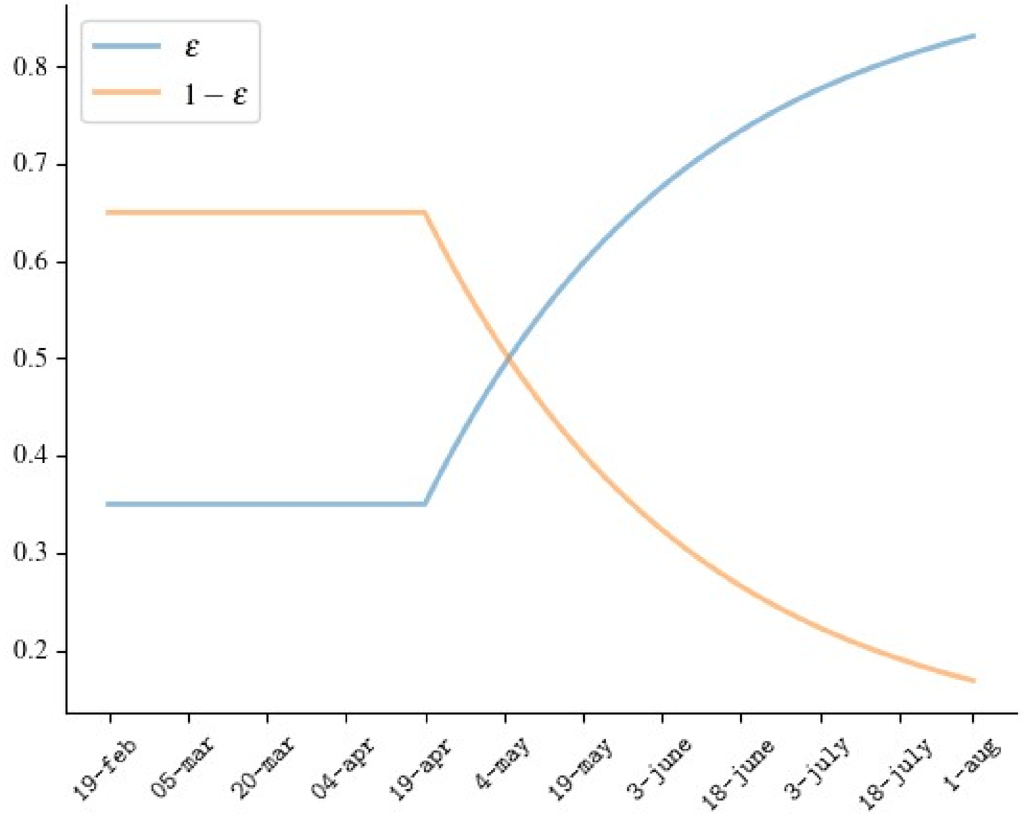

S3 Fig. Time evolution of the asymptomatic fraction ε(t).

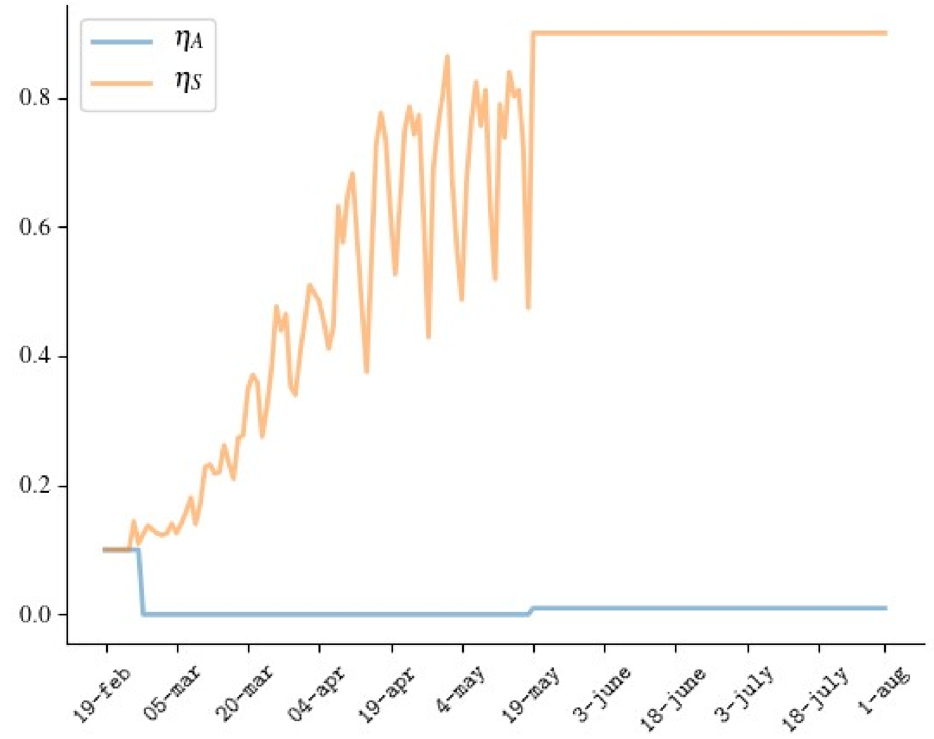

S4 Fig. Time evolution of the detection rates $\eta_{A,S}(t)$.

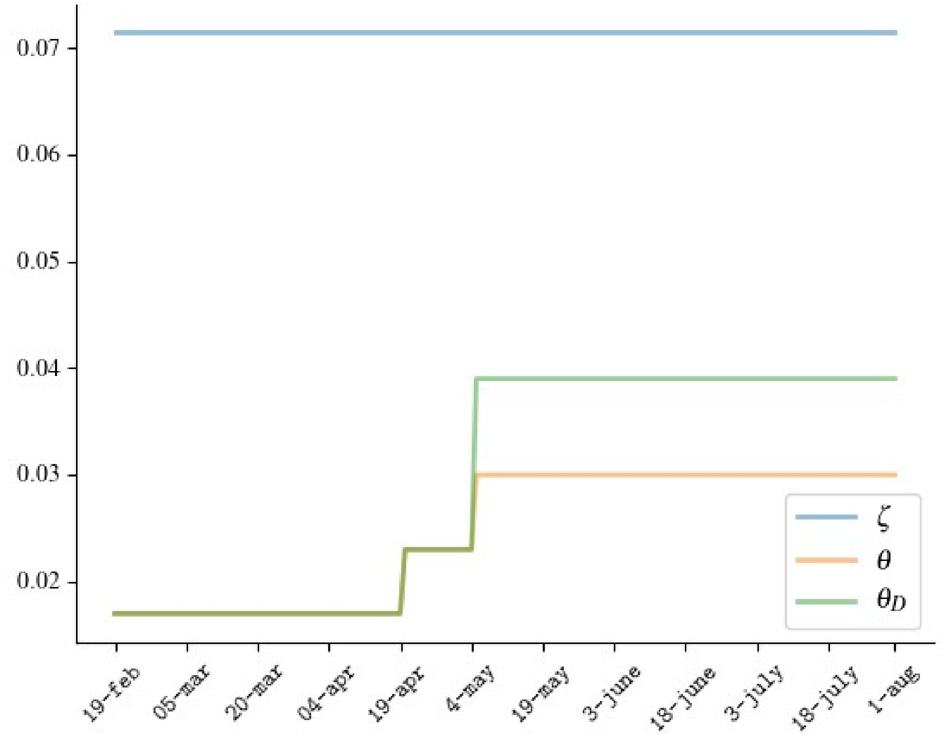

S5 Fig. Time evolution of the healing rates ζ, θ, $\theta_D(t)$.
